# High-Throughput *In Vivo* Genotoxicity Testing: An Automated Readout System for the Somatic Mutation and Recombination Test (SMART)

**DOI:** 10.1371/journal.pone.0121287

**Published:** 2015-04-01

**Authors:** Benoit Lombardot, Chun-Taek Oh, Jihoon Kwak, Auguste Genovesio, Myungjoo Kang, Michael Adsett Edberg Hansen, Sung-Jun Han

**Affiliations:** 1 Image Mining Group, Institut Pasteur Korea, Sampyeong-dong 696, Bundang-gu, Seongnam-si, Gyeonggi-do, Korea; 2 Drug Biology Group, Institut Pasteur Korea, Sampyeong-dong 696, Bundang-gu, Seongnam-si, Gyeonggi-do, Korea; 3 Department of Mathematical Sciences, Seoul National University, 1 Gwanak-ro, Gwanak-gu, Seoul 151–747, Korea; National Cancer Institute, UNITED STATES

## Abstract

Genotoxicity testing is an important component of toxicity assessment. As illustrated by the European registration, evaluation, authorization, and restriction of chemicals (REACH) directive, it concerns all the chemicals used in industry. The commonly used *in vivo* mammalian tests appear to be ill adapted to tackle the large compound sets involved, due to throughput, cost, and ethical issues. The somatic mutation and recombination test (SMART) represents a more scalable alternative, since it uses *Drosophila*, which develops faster and requires less infrastructure. Despite these advantages, the manual scoring of the hairs on *Drosophila* wings required for the SMART limits its usage. To overcome this limitation, we have developed an automated SMART readout. It consists of automated imaging, followed by an image analysis pipeline that measures individual wing genotoxicity scores. Finally, we have developed a wing score-based dose-dependency approach that can provide genotoxicity profiles. We have validated our method using 6 compounds, obtaining profiles almost identical to those obtained from manual measures, even for low-genotoxicity compounds such as urethane. The automated SMART, with its faster and more reliable readout, fulfills the need for a high-throughput *in vivo* test. The flexible imaging strategy we describe and the analysis tools we provide should facilitate the optimization and dissemination of our methods.

## Introduction

Genotoxicity can occur from a chemical compound causing damage to the genetic material, ultimately resulting in disease and/or death. Testing for genotoxicity represents an important part of meeting the toxicity regulations and recommendations, which are becoming more and more common across various industries [1–3] including pharmaceutical, cosmetic, and automobile manufacturing industries, among others. These regulations were created to ensure the safety of the public as well as workers exposed to various chemical agents involved in the manufacturing process of different products. Toxic side effects, including genotoxic ones, are a major reason for the delay or termination of drug development [4–5]. Consequently, compounds undergo a number of complementary *in vitro* and *in vivo* tests to enable the prediction of such effects [6]. The latter class of tests involves small animals such as rodents. In addition to the ethical problems associated with research in mammals, these tests can be quite costly and time consuming [7]. For this reason, *in vivo* tests are usually carried out after or just before the selection of the lead compound.

**Fig 1:**
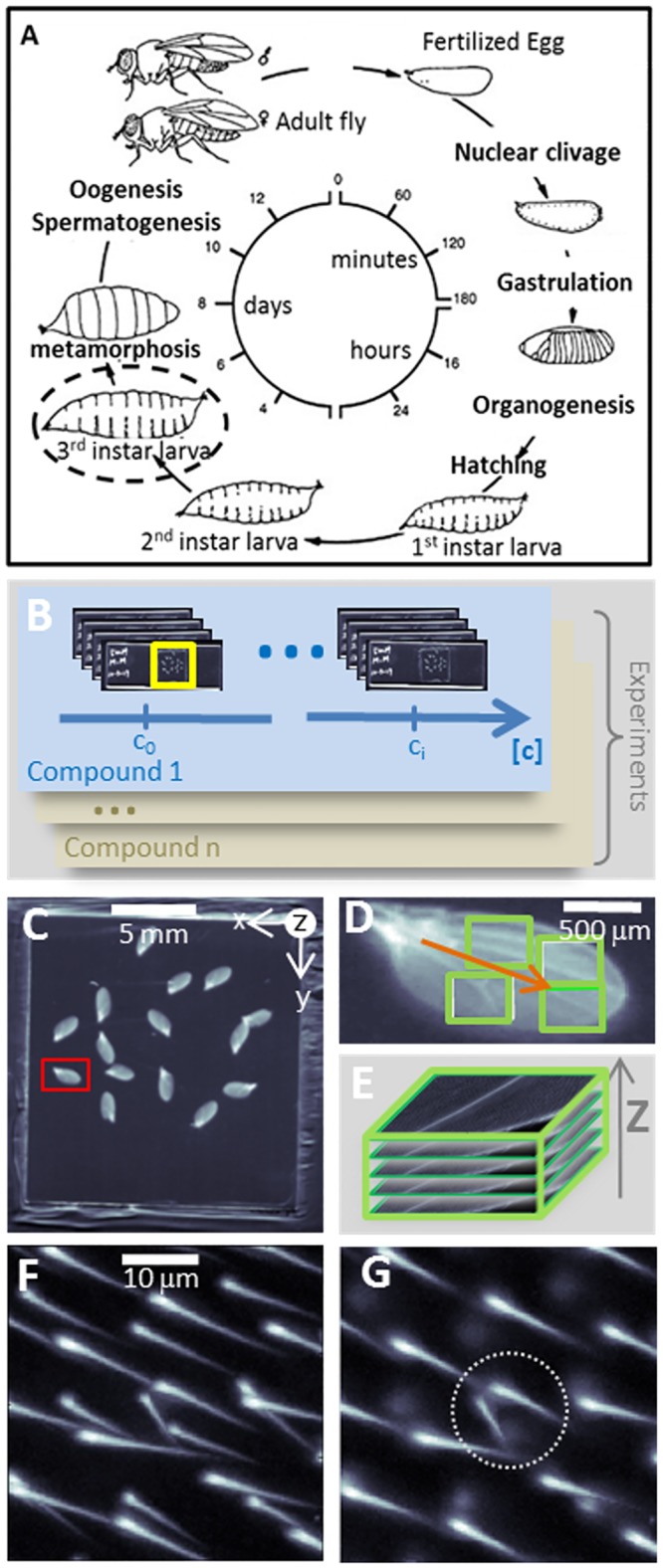
SMART automated image and data acquisition. To test chemical-induced genotoxicity, fly larvae (A) were exposed to increasing doses (B; c0, ci, for instance) of a test compound and their wings (C), along with those of other flies from their treatment group, were collected on a slide. A single wing is shown in the red rectangle. (D) Wing position and orientation (orange arrow) were detected automatically, and acquisition regions were defined (green rectangles, each corresponding to a microscope field of view). All acquisition positions defined were compiled in a single file used by the microscope to perform multipoint acquisitions. (E) At each point, a focus stack showing wing hairs and their spatial organization was acquired. (F) A close-up of a focus stack maxima projection along the focus axis, showing hair organization. Hairs from the upper and lower sides of the wing overlap in this view. (G) For illustrative purposes, lower wing hairs have been separated by manually selecting a set of z-slices before projection. Focusing on a single wing side, one can distinguish the regular position and orientation of the hairs. Hairs with an *mwh* phenotype are visible (white dashed circle).

Introducing a faster, more scalable test that can be performed early in drug development is a major focus of interest, as it would help allocate resources to the most promising candidate compounds. Due to their quick reproductive cycles, greater ethical acceptance, and smaller need for infrastructure, small, non-mammalian animals, such as round worms (*Caenorhabditis elegans*), zebrafish (*Daniorerio*), and flies (*Drosophila melanogaster*) are good candidates for the development of high-throughput genotoxicity tests. In particular, the Somatic Mutation and Recombination Test (SMART), also known as the wing-spot test, assesses the loss of heterozygosity (LOH) resulting from genotoxicity [8–10].In this test, flies carrying heterozygous recessive mutations for the multiple wing hair (*mwh*) and flare (*flr*) phenotypes are exposed to a test compound. Visual inspection of wing hair phenotypes in adult flies allows characterization of LOH (Fig 1). SMART has also been shown to detect genotoxicity associated with metabolized chemicals [11–12], as well as anti-genotoxic effects [13–14].

Thus far, the use of SMART has involved small compound sets due to throughput limitations that restrict the size of the studied cohorts. The visual inspection of hair phenotypes is a time-consuming and tedious task, prone to fatigue error and inter-expert variability. Providing an automated method for examining wing hair phenotypes would allow us to increase the throughput of SMART while improving its reliability, facilitating the study of dose-response relationships.

The *Drosophila* wing is organized into seven regular sectors, delineated by veins. As observed using bright-field microscopy, a wild-type hair presents as an elongated structure, which is thinner at the root than at its tip, laying at an acute angle to the wing surface. Hairs cover the entire upper and lower surfaces of the wing, with locally uniform orientation, size, and inter-hair distance. In the SMART, genetic recombination can create cells homozygous for the *mwh* or *flr* mutations, resulting in specific phenotypes, i.e. *mwh*, *flr*, or more generally mutant phenotypes. An *mwh* cell is characterized by close-growing multiple hairs, typically two to four, of various lengths. The *flr* phenotype involves a single shortened and amorphous hair. Due to the cell division, which occurs during the development of the fly, mutant cells often form a cluster of clones that display similar phenotypes, termed as a mutant spot. The SMART exploits the increased rate of mutant spots, which occurs in the presence of a genotoxic compound, to identify genotoxicity.

Automated scoring is common for many genotoxicity tests, including the micronucleus [15] and comet tests [16]. However, the automation of the SMART has not yet been reported. Automated detection of hair phenotypes has not been studied, despite its potential applications in genotoxicity tests, developmental research, and cell polarity studies [17–19]. However, many well-established methods exist for systematically detecting biological objects, such as nuclei [20–22], constituting potential approaches for wing hair detection and SMART analysis. Methods to detect entangled rod-like structures in low signal environments [23] and various tubular shapes [24]have also been demonstrated. No method has been described for detecting *mwh* or *flr* phenotypes or identifying mutant spots thus far. Moreover, the sensitivity of any method will have to be evaluated against the spontaneous rate of incidence of mutant hairs in control wings, which has been reported as 0.2 *mwh* hairs per fly [8].Omitting one of the mutations from the SMART could help improve its accuracy by simplifying the analysis required, since a single mutation may be sufficient for assessing the genotoxicity, although we lost the function to deduce what kind of recombination is happened through analyzing of twin spots.

Bright-field microscopy allows researchers to obtain an image of hairs with good contrast. Though it is possible to visualize an entire *Drosophila* wing using a low-magnification lens, the resolution obtained is not sufficient to detect individual mutant phenotypes at the hair level. The resolution of micron-wide hairs necessitates the splitting of the wing image acquisition into several parts. Furthermore, the fact that the wing is not flat and has hairs on both sides suggests that the acquisition of focus stacks is the correct approach. Focus stacks would simplify hair analysis by providing images without overlapping objects, as well as providing a natural separation of upper and lower wing hairs. If necessary, bright-field confocal imaging could be used to improve the axial resolution.

Finally, to prevent image acquisition from becoming a bottleneck, the wing image acquisition should be automated. While automatic whole slide imaging solutions exist [25], the time and storage demands associated with these methods limit their potential. In addition, it is necessary to detect the wing position on the slide images following the image acquisition. Within our proposed method, the acquisition positions are predetermined using a low-resolution image of the slide, allowing the researchers to automate slide acquisition using a microscope equipped with a motorized stage. This approach could save precious time and minimize data storage requirements. To the best of our knowledge, such a method has not yet been demonstrated for this application.

**Fig 2:**
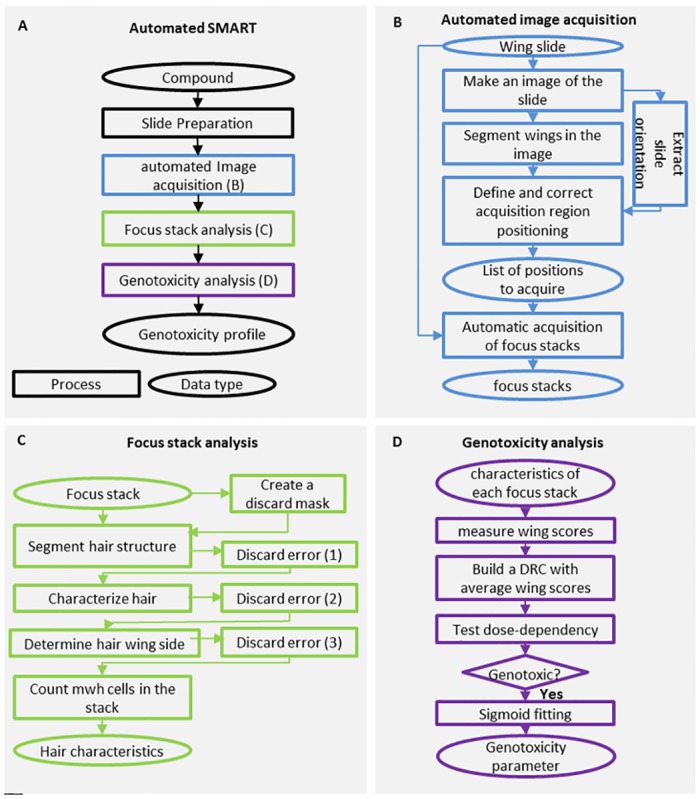
Automated SMART process. The methods used to automate the SMART are depicted with a flow diagram. The general flow of the method is described in (A). Each step in the process is depicted by a rectangle, with data inputs and outputs depicted using elliptical shapes. (B) Steps in automated acquisition. (C) Steps in focus stack analysis and the extraction of data on *mwh* hairs, cells, and spots. Discard error (1–3) refers to detection of (1) abnormal hair shape, (2) abnormal hair orientation, and/or (3) abnormal position relative to the wing surface. (D) Steps in the construction of the genotoxicity wing score dose-response curve and the characterization of compound genotoxicity.

In this paper, we propose an automated SMART, a four-step process for characterizing a compound’s genotoxicity (Fig 2). First, fly larvae carrying one copy of the *mwh* mutation are exposed to a range of doses of the test compound. Second, each slide is automatically processed to determine the positions at which the microscope will acquire the images. Focus stacks are then acquired, with no human intervention required outside of individual slide acquisition initialization. Third, each focus stack is analyzed with a dedicated image-processing algorithm to detect *mwh* hairs. Finally, a genotoxicity score is calculated for each wing and the dose-dependency of scores is analyzed to characterize a compound’s genotoxicity. This automated process has been applied to six compounds with known genotoxicity profiles to compare its performance relative to manual scoring.

## Results

### Automated imaging performance

Automated imaging encompassed both slide preprocessing and image acquisition (Fig 1C–1E).Using this method, images of more than 3,000wingswere acquired, with an average of more than five focus stacks per wing.

Automated acquisition quality is assessed by (1) specificity, or a method’s ability to image a region of interest, and (2) coverage, or its ability to image all of a region of interest. Specificity decreases when a region outside of the wing is imaged. We calculated a specificity level of 97% and a coverage level of66%. These numbers do not take into consideration for the area accounted for by veins, since this cannot be analyzed after slide preprocessing. The manual correction of wing segmentation, requiring only5 minutes per slide, played an important role in attaining the high specificity. Nevertheless, the definition of some images was compromised due to suboptimal detection of slide orientation, though this effect was limited.

Identification of regions of interest during slide preprocessing allows a researcher to efficiently navigate these areas under the microscope. We found that the determination of relative position of the predefined regions was very accurate. Absolute position, however, can be corrected manually by moving the XY stage to the initial position, after which acquisition proceeds automatically. The entire acquisition process, including slide preprocessing, required 1 hour and 25 minutes, with only 10 minutes involving manual manipulation. This process provided images with good contrast. In particular, the axial resolution appeared to be sufficiently high to separate hairs without the need for confocal microscopy.

### Hair detection and characterization

Automated analysis of focus stacks allowed the detection and characterization of more than13 million hairs (an average of 4,246 hairs per wing). Of these, 0.15% expressed the *mwh* phenotype (an average of 6.37*mwh* hairs per wing), with the prevalence on a single wing varying from 0 to 8.9%. The number of hairs that were incorrectly identified as *mwh* (false positives, or FPs) was greater than the number of *mwh* hairs that were not detected (false negatives, or FNs). FPs were measured in a subset of slides: those from larvae exposed to methyl methanesulfonate (MMS) and those from control larvae (see S1 File). An average of 0.43±0.23 FPs were detected per control wing, a number significantly below the average number of *mwh* hairs detected. Moreover, the number of FPs observed was not significantly different between different MMS concentrations (*P* = 0.06), suggesting that the FP rate is not dose-dependent. In control wings, variation in the number of FPs accounted for only 10% of the variance in *mwh* hair counts among the samples. Measurement error therefore does not appear to be masking genotoxic effects (see S1 File). FNs were assessed qualitatively, by visual inspection. We found that our method performed very well in detecting isolated *mwh* phenotypes. The number of FNs increased in large clusters of *mwh* cells, however, due to the higher complexity of the patterns observed.

**Fig 3:**
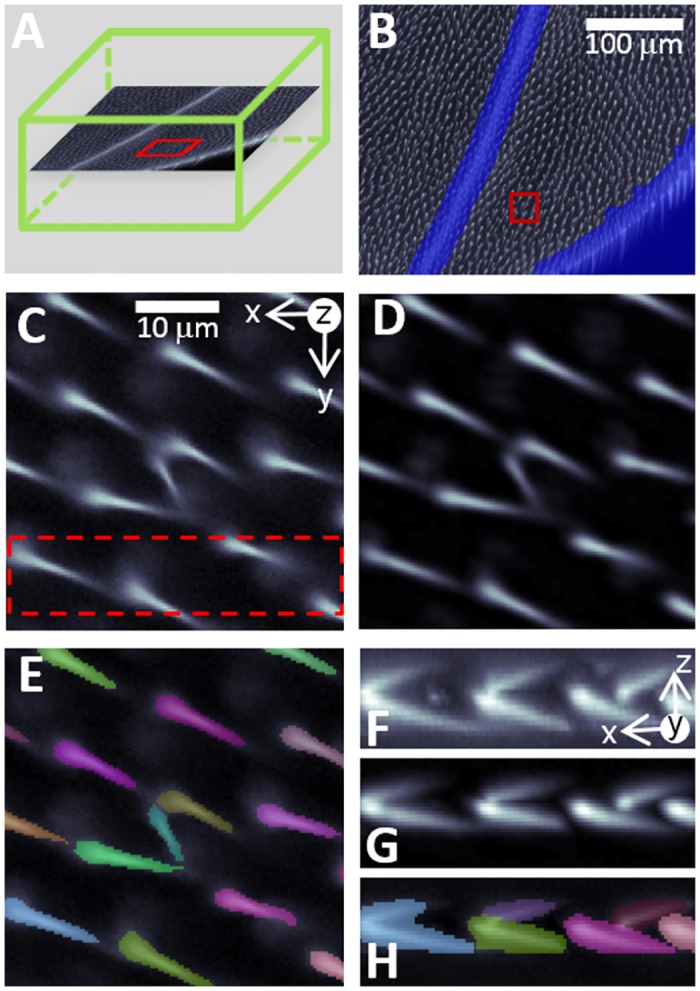
Wing hair segmentation. Data on wing hairs were extracted from each focus stack, which covers a subsection of the wing. (A) Focus stack volume is shown in green, with one z-slice displayed. (B) Unwanted regions (veins, wing borders) are detected and discarded prior to hair analysis. A typical selection is shown, with a blue overlay on top of the z-stack projection. A close-up of a z-slice (A, red rectangle) is used to illustrate (C) the original image details, (D) filtered image details, and (E) a segmented hair region with a random color overlaid on top of the filtered image. A selection [red, dashed rectangle in (C)] is shown in lateral view (maximum projection of the sub-volume against the y-axis) in (F, G, and H), illustrating the original, filtered, and segmented data, respectively. The white scale in (C) indicates the dimensions along the x and y-axes in images (C–H).

The image analysis process is illustrated in Fig 3. Discarding veins and background areas appears to be a very important task, since failing to do so resulted in a 12-fold increase in *mwh* detection, whereas the total number of hairs analyzed increased by only 34%. This suggests high detection of FPs in removed regions, an observation verified by visual inspection. The discard mask we defined was very effective, only rarely missing small sections of veins or wing borders. Although this could lead to detection of FPs, it appeared to be only a minor source of error in our data. Furthermore, the discard mask was usually wider than the region discarded, implying a limited rejection of areas that could have been analyzed.

Hair segmentation did not prove to be particularly problematic, thanks to good contrast and well-separated hairs. The filtering process we used was simple and computationally efficient, while providing a uniform background for segmentation. Deconvolution filter was not needed, since individual hairs were well separated in the axial direction. The use of h-maxima efficiently dispatched multiple local maxima in hair structures, avoiding over-segmentation. Under-segmentation can occur when hairs have roots that are very close to one another. In the final steps of the analysis, 7.3% of segmented objects with unusual shape descriptor values were discarded (see Methods). However, their effect on *mwh* hair counts was low; failing to discard them changed the average number of *mwh* hairs detected per control wing from 1.44 to 1.84. Feature-based segmentation [26–27] or region grouping could potentially improve segmentation quality by automatically eliminating objects with unusual shapes.

**Fig 4:**
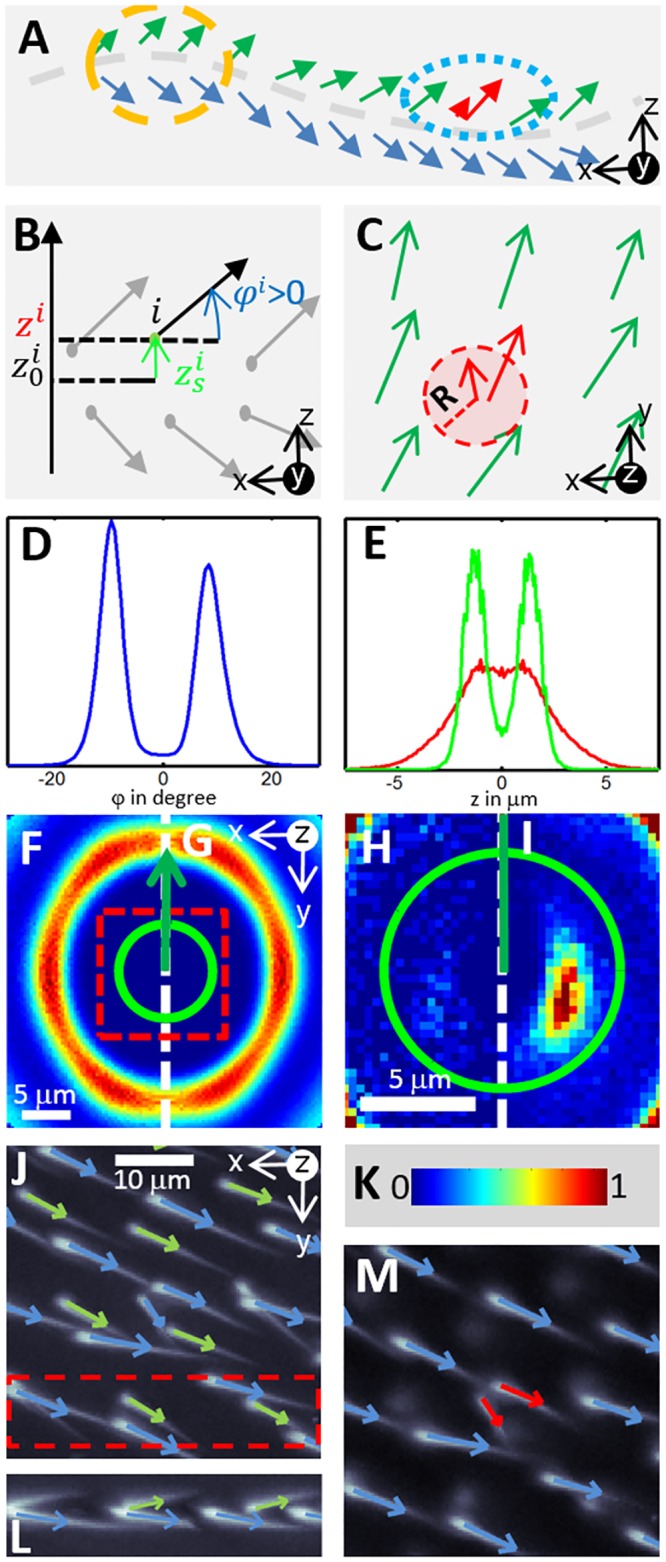
*mwh* hair detection. All segmented hairs are characterized by vectors prior to the detection of *mwh* phenotypes. (A) A schematic side view of the wing illustrates hair organization, with hairs on each side of the wing surface (dashed gray line): top (green) and bottom (blue). A close-up of this schematic (A, orange circle) illustrates that (B) locally, the wing can be considered flat. Its altitude (z_0_) can be estimated from local hair roots. Hair root altitude relative to the surface (z_s =_ z-z_0_) and angle relative to the surface (*φ*)are used to determine the wing side. Indeed, both the *φ* distribution (D) and the z_s_ distribution (E, green curve) present two modes corresponding to top and bottom hairs. Absolute hair root altitude (z) distribution (E, red curve) does not show such a separation. (C) presents a bird’s eye view of hair organization on the top side of the wing. Detection of *mwh* hairs relies on their small inter-hair distance (red circle and arrow). The distribution of relative positions of adjacent hairs illustrates this characteristic. (F–G) show distributions built from control and total samples, respectively. (H–I) show details of these distributions. These distributions adhere to the color map in (K). Only their left or right side is shown, due to distribution symmetry, and their highest density is set to one in each image, allowing more details to be seen in (H–I). Distributions may look similar, but their close-ups are distinct; the distribution determined in total samples has a specific mode that corresponds to the *mwh* hair phenotype. A distance threshold (green circle) allows us to select *mwh* hairs using that mode. (J) An example of hair wing side detection (bottom hairs in blue, top hairs in green) is overlaid on a focus stack detail. (L) A side view of the section of (J) is presented in the red, dashed rectangle. (M) An example of *mwh* hair detection (red arrows) is overlaid on a manually constructed image of wing bottom hairs.

The principles of hair characterization are illustrated in Fig 4. Detecting hair origin, orientation, wing side, and phenotype proved to be straightforward. Positioning the hair root at the highest intensity point of each segmented region was very reliable, and the rare mispositioned hair could be discarded by verifying that adjacent hairs had dissimilar orientations. In practice, 0.3% of the hairs were discarded in this way. The distribution of hair angles (Fig 4D) and hair root altitudes (Fig 4E) relative to the wing surface exhibited two distinct modes, corresponding to hairs on the top and bottom side of the wing. The best separation was obtained using the angle of individual hairs; however, the addition of altitude allowed inconsistent patterns to be detected and discarded (see Methods). Finally, the detection of an *mwh* hair was based on the distance between that hair and its nearest neighbor. The threshold distance used was an accurate mean of discrimination, as can be seen from the distribution of the relative positions of adjacent hairs (Fig 4F–4I). In general, all parameters used in the analysis could be deduced from the images or hair characteristics. This should allow the presented results to be easily reproduced.

### Application of SMART automation

Our automated SMART approach was applied to the analysis of six test compounds. Three of them, mitomycin C (MMC), MMS, and urethane (Ur), are known genotoxins with high, moderate, and low-level genotoxic effects (MMC>MMS>Ur). The three other tested chemicals are non-genotoxic compounds isoniazid (Iz), antipyrin (AP), and atenolol (At). Each compound was analyzed across a range of doses. Our automated method was used to calculate the wing genotoxicity scores. Manual wing scores were also calculated, to aid in assessing the results from the automated SMART. Additionally, if a compound displayed excessive toxicity at a certain dose, a lower concentration was substituted (see S1 File). For this reason, the original tested concentrations of MMC (2.5, 5, and 10 mM) were replaced with 0.04, 0.08, and 0.16 mM concentrations.

### Genotoxicity characterization

Our genotoxicity measure is based on dose-dependent wing genotoxicity scores. It differs from the classical SMART characterization of genotoxicity [8]in terms of the types of scores measured and the statistical tests employed [28].

The sensitivity of different genotoxicity scoring methods was investigated (see S1 File). Scores based on *mwh* cell counts were best suited for the assessment of genotoxicity, since they were less prone to FPs than spot counts. Indeed, we found the spot count score to be less sensitive to compounds that cause the formation of few large spots rather than many small spots. In our analysis, the genotoxic wing score refers to a number of *mwh* cells per wing.

We subsequently tested whether the average score at each concentration differed from the control score (see S1 File). This two-sample test is less stringent than the doubling effect test, but its success is a prerequisite for the success of the doubling effect test. Additionally, we assessed whether the effect of each compound on the wing scores was dose-dependent (see S3 Table). MMC, MMS, and Ur were identified as genotoxic, while the other three compounds tested negative. These results are in accordance with the known genotoxicity profiles of the tested compounds.

In the original SMART, genotoxicity was assessed using a doubling of the genotoxic effect relative to the control as a criterion. However, this method has a number of limitations: possible inconclusive tests, low sensitivity in the presence of an offset in the genotoxicity wing score, and sensitivity to outliers. The dose-dependency test is a good candidate for addressing these issues. It operates independently from the score offset and is more robust to outliers, as it combines all dose data. We found the dose-dependency test to be more sensitive at detecting genotoxic compounds than the two-sample test. Notably, Ur, a compound with low genotoxicity, was not identified as genotoxic using the two-sample test at the concentration range initially tested (0.3–10 mM) with both manual and automated measures. Conversely, the dose-dependency test was positive at that range. The results of the two tests were in agreement for other tested compounds.

**Fig 5:**
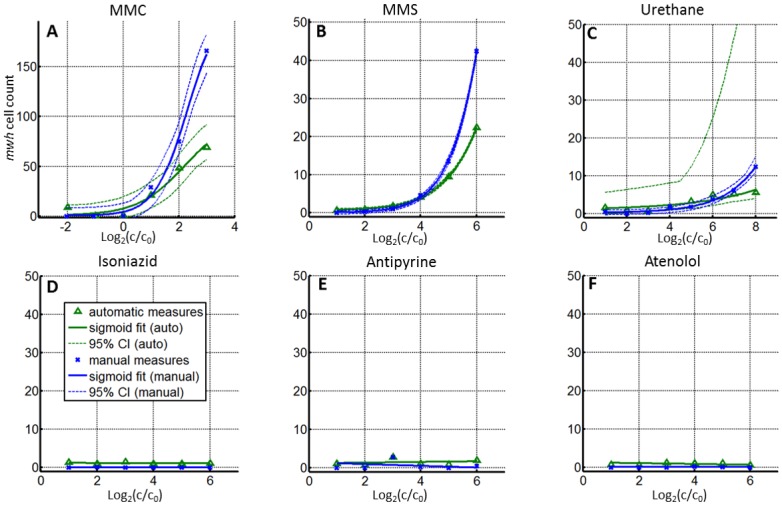
Genotoxicity analysis. DRCs for 6 test compounds. Manual measures (blue crosses) and automated measures (green triangles) were obtained, allowing us to detect genotoxic (A, B, and C) and non-genotoxic (D, E, and F) compounds. Known genotoxins (first row) all exhibited increasing genotoxic effects with increasing concentration. Further analyses with fitting a sigmoidal curve to the data (A, B, and C; solid lines) and 95% confidence envelopes (dashed lines) allowed the EC_50_ and slope parameters to be determined, facilitating characterization of compound genotoxicity. Findings from manual and automated measurements were highly correlated after scaling. Non-genotoxic compounds (second row) showed no significant dose-dependence (linear fit shown as a solid line) in either manual or automated measures.

Genotoxic compounds were further analyzed by fitting the sigmoidal curve to the data. The fitting parameters described experimental and genotoxic compound characteristics. Range parameters accounted for the scaling between different measures on a same wing, while bottom parameters described the offset between the two measures. The remaining parameters, EC_50_ and slope, were related to the concentration at which 50%of the maximal effect occurred and to the magnitude with which the effect was increased by increasing dose, respectively. These two parameters provide a simple characterization of genotoxicity that makes use of all dose-dependent measures to yield information not provided by the original SMART. Average wing score values and curve fittings are shown in Fig 5 and Table 1. Fitting of the sigmoidal curve was performed for MMC, MMS, and Ur by enforcing an adequate range of concentrations (choice of concentrations is explained in S1 File). The EC_50_ and slope values obtained from both manual and automated measures were in accordance with the known genotoxic characteristics of the compounds(MMC>MMS>Ur). EC_50_ confidence intervals did not overlap between the compounds. Conversely, slope confidence measures were less accurate discriminators. Since the genotoxic effect of Ur appeared to be low, two additional concentrations (20 and 40 mM) were tested to obtain its genotoxicity profile. However, it still exhibited greater EC_50_ and slope variability than MMC or MMS.

**Table 1:** Genotoxicity data for six test compounds.

**Compound**	Measure type	Dose dependency sign(P-value)	EC_50_(mM)	EC_50_95% CI (mM)	Slope	Slope 95% CI
**MMC**	**Auto**	+ (1 × 10^-3^)	0.75	[0.51, 1.11]	1.15	[0.69, 1,91]
**MMS**	**Auto**	+ (4 × 10^-3^)	24	[23, 26]	1.03	[0.97, 1.10]
**Urethane**	**Auto**	+ (3 ×10^-3^)	1.1 × 10^4^	[23, 5.5 × 10^6^]	0.17	[0.01, 2.39]
**Isoniazid**	**Auto**	- (0.66)	_	_	_	_
**Antipyrine**	**Auto**	- (0.35)	_	_	_	_
**Atenolol**	**Auto**	- (0.89)	_	_	_	_
**MMC**	**Manual**	+ (3 × 10^-3^)	0.76	[0.67, 0.85]	1.74	[1.37, 2.20]
**MMS**	**Manual**	+ (6 × 10^-3^)	21	[20, 22]	1.28	[1.21, 1.35]
**Urethane**	**Manual**	+ (3 × 10^-3^)	6.8 × 10^2^	[310^2^, 1.3 × 10^3^]	0.68	[0.54, 0.86]
**Isoniazid**	**Manual**	- (0.36)	_	_	_	_
**Antipyrine**	**Manual**	- (0.78)	_	_	_	_
**Atenolol**	**Manual**	- (0.69)	_	_	_	_

Data were obtained using both automated and manual measurements. Presented findings are based on the number of *mwh* cells per wing.

### Comparison of automated and manual measures

Excluding sample preparation time, analysis of a single slide took 2 hours and 10 minutes using our automated process, including 10 minutes for manual imaging manipulation, 75 minutes for acquisition, and45 minutes for the detection of *mwh* hairs. Since each compound tested required a total of 28 slides, less than 5 hours of manual work was required. This is an eight-fold reduction in operating time, compared to the length of time required for equivalent manual genotoxicity analysis.

The results of the dose-dependency tests were the same for manual and automated measurements and matched our knowledge of the genotoxicity of the tested compounds. In particular, low-genotoxicity of Ur was easily detected using the initial range of concentrations tested (0.3–10 mM).

Manual and automated wing scores could not be directly compared due to protocol differences involving the analyzed wing surface area and error rates. However, sigmoid range and bottom parameters were used to account for these differences, allowing us to compare theEC_50_ and slope parameters obtained from both methods (see S1 File). EC_50_and slope values from the fitted curves obtained using this scaling process are presented in Table 1. These values were obtained using wing scores based on *mwh* cell counts. Slope values obtained using automated measurements had a tendency to be smaller than the values based on manual measurements. This could be explained by the relatively higher number of FNs observed using the automated analysis under the most genotoxic conditions. EC_50_valuescalculated using either automated or manual measurements were almost identical for MMC and MMS. Those calculated for Ur were also similar, despite the relatively larger confidence intervals in the data obtained using automated measurements. The difference observed between manual and automated Ur wing scores seems to reflect the heterogeneous spatial distribution of *mwh*cells. In particular, mutant cells apparently occurred closer to the base of the wing, which was not analyzed using the automated process.

## Discussion

Genotoxicity is an important consideration in toxicity regulations. Introducing a faster, more scalable, *in vivo* test would benefit public and worker safety in all sectors of industry, as it would allow more systematic and reliable testing of the chemicals with which we come into contact daily. With that aim, we propose a method to automate the SMART. In addition to simplifying the genetic basis of the test, we have introduced an automated imaging process, thus eliminating the image acquisition bottleneck. We have also developed an image analysis pipeline that detects *mwh* hairs in wing focus stacks, generating genotoxicity scores for each wing. Finally, we propose a dose-dependency analysis that yields an informative genotoxicity profile for each tested compound.

We validated the proposed automated methods by comparing automated and manual measurements for six test compounds, obtaining almost identical genotoxicity profiles using the two methods. Automated measurements proved to be sensitive enough to identify compounds with low genotoxicity, such as Ur. Additionally, the genotoxicity profiles we obtained with automated measurements perfectly matched the expected toxicity for all test compounds. Moreover, the results were obtained much faster. The time required was reduced eight-fold relative to the time required for manual analysis, from one week to five hours, resulting in the increase of the SMART throughput. We also found that *mwh* hair and cell counts provided a reasonable alternative to *mwh* spot counts for the SMART. Finally, the dose-dependency analysis developed here allows more sensitive and detailed characterization of genotoxicity than the two-fold increase test commonly used.

This method provides an elegant and promising solution that meets the need for a fast and accurate *in vivo* technique for assessing genotoxicity, one that is ethically acceptable and easy to carry out. Automation allows analysis of a larger cohort of compounds and avoids the high variability inherent to manual measurements, ensuring more reliable results than those obtained by manual methods. We provide a MATLAB toolbox with custom code for each step of the analysis (slide preprocessing, *mwh* hair detection, and genotoxicity characterization). Image acquisition can easily be adapted to any microscope equipped with a computer-controlled stage. Overall, the proposed method requires only standard, affordable instruments. We believe that the materials provided and methods described will provide a starting point for an automated SMART. Improvements may emerge over time, including faster and more exhaustive wing image acquisition, more sensitive hair phenotype detection, and the ability to recognize additional hair phenotypes.

## Methods

### Fly and sample preparation

This study was conducted with *Drosophilamwh* ^1^ (*mwh* ^1^/ *mwh* ^1^) and *w* ^*1118*^ fly strains obtained from the Bloomington *Drosophila* stock center, prepared according to the following procedure: virgin *w* ^*1118*^ females and *mwh* ^1^ males were mated in a culture bottle for 8 hours at 25°C,with yeast cornmeal food. We obtained 200–300 fertilized eggs carrying the heterozygous marker (*mwh* ^1^ /+), which were kept in the same media for 72hours, after which larvae were washed with distilled water and treated with different concentrations of the test compounds (0, 0.3, 0.6, 1.25. 2.5, 5, or 10 mM). Test compounds were mixed with 10% dimethyl sulfoxide (DMSO) medium and previously washed larvae. After 6 hours of exposure, roughly70–100 larvae per compound/dose were transferred to yeast cornmeal food. Experiments were carried out at 25°C with 60% humidity and a 12-hour light/dark cycle. The ratio of hatched flies was measured, and hatched male flies were collected and stored in 70% ethanol. More than 60 wings per compound/dose, one per fly, were mounted on slides (about 15 wings per slide) in Faure’s solution [10], and *mwh* mutant wing hairs were analyzed manually under 200× magnification [8].

Details on DMSO toxicity and genotoxicity are provided in the S1 File.

### Software and computation tools

All data analyses were performed using custom MATLAB R2013a (Mathworks) software on a computer with an Intel core i7 3970X processor, 24 GB RAM, and a Windows 8 64-bit Professional operating system. The hair segmentation analysis, in particular, was performed with a custom-made watershed algorithm implemented in C# and compiled to a dll file accessed inMATLAB. Alternatively, MATLAB watershed function (in the image processing toolbox) can be used at the expense of longer processing time. We provide the MATLAB scripts and algorithms developed for the SMART automated readout, with further description of its contents provided in the S1 File. The ND2 file generated by our Nikon software is read using Nikon ND2 SDK library, distributed by Nikon upon request.

### Slide preprocessing for image acquisition

Each sample slide was preprocessed to determine a list of acquisition positions. We determined multiple positions for each wing on a slide as follows: A low-resolution, full image of the slide was acquired with a standard desktop scanner (Epson perfection V33), using Epson Scan software at 300 dpi, 8 bit grayscale. A threshold of 15% of the intensity range was applied to the inverted image to select the wing regions. In an optional step, the mask obtained was filtered to regularize its contours and improve the separation of wing area. A labeling filter was then applied to the filtered mask to identify the distinct regions. Regions that did not conform to normal wing parameter ranges were discarded. The parameters considered included region aspect ratio, area, and average position. Normal wing position was defined by manual selection of the lamella region. Other parameter ranges were defined as parameter averages± 2× their standard deviation, as determined from manual measurements in test images. In particular, the region aspect ratio was calculated from an individual region’s pixel position covariance, as$\sqrt{\lambda_{1}/\lambda_{2}}$, where λ_1_ and λ_2_represent the first and second eigenvalues of the covariance matrix and a region’s area was defined by the number of pixels it encompassed.

In the second step, a wing‘s origin region was removed, since no images needed to be acquired there. The origin was defined as the position of maximum intensity. Prior to maximum detection, a local blurring inside the wing region improved the robustness of detection. An axis was subsequently defined with an origin at the wing origin and orientation determined by a vector going from the origin of the wing to the center of mass of the wing. Regions (1) with a distance to origin length shorter than 400 mm, i.e. roughly 20% of average wing length ($\sqrt{\lambda_{1}}$) or (2) on the negative side of the wing axis, were not analyzed in the steps that follow. In cases in which wing detection and characterization were problematic, wing segmentation and origin were checked manually and, if necessary, updated using a custom-made MATLAB interface.

Each of the wing regions was then partitioned with a k-mean classifier [29] using the wing’s pixel positions as input. The average position of each class defined an acquisition position for the microscope. The number of classes was optimized to limit the overlap between regions of acquisition. To account for wing area variability, the number of acquisitions decreased in proportion to the wing area. For instance, defining a maximum of six partitions would lead to coverage of 67% of the wing surface.

Next, the positions defined using the slide image had to be translated into microscope coordinates. Using a fixed sample holder, the slide’s edges were aligned to the microscope XY stage displacement axes. Automated detection of the slide center (*X*_*c*_) and orientation (*ϑ*) allowed us to determine the microscope referential from the slide image (see S1 File). Image acquisition position *X* could thus be translated to the microscope coordinates *X*' using the following formula:

$$\;X^{\prime }=\frac{r_{mic}}{r_{img}}\;.R_{\vartheta }\left(X-X_{c}\right),$$

where *R*_*ϑ*_ is a rotation transformation of angle ϑ and $\frac{r_{mic}}{r_{img}}$is the ratio of microscope planar sampling to slide image sampling.

### Image acquisition

Image acquisition was performed using bright-field microscopy with a Nikon eclipse 90i transmission microscope equipped with a Nikon DS-1QMcooled to -30°C, sensitive black and white charge-coupled device (CCD), 1280 × 1024 pixel camera, and a Nikon APO Plan 20X, 0.75NA, DIC N2 WD 1.0lens. System planar sampling was performed at 0.32 micron per pixel, while the axial sampling resolution was 1 micron. Samples were repositioned with a motorized XY stage and a Z focus. The system was controlled with Nikon NIS Element AR software version 3.2, which includes a built-in interface for z-stack and multipoint acquisition. Z-stack parameters and a precalculated list of positions were loaded to the “ND experiment” module of the NIS Element software. The microscope was then manually initialized at the first position to be acquired. The rest of slide acquisition was fully automated; the sample was automatically moved to each predefined position, where an automated focus was performed, followed by z-stack acquisition. The spatial volumes acquired were saved to the Nikon ND2 file format for further analyses.

### *mwh* hair detection

The elongated shape of the hair of the wild-type and *mwh* phenotypes allowed us to segment and characterize these hairs in a similar manner.

Top-hat filtering followed by Gaussian blurring enabled us to adjust for a slowly varying background resulting from an out-of-focus signal or noise in the z-stack, respectively. Parameters were chosen to avoid creating hair structure artifacts. The Gaussian standard deviation and top hat filtering radius were set to values equal to the fraction of a hair width (2 μm) and a value larger than a hair width, respectively.

Image intensity local maxima were subsequently used to detect hair positions and initiate watershed segmentation of the image, allowing the creation of a labeled image of the hair region. In watershed segmentation, distinct labels are given to each maximum. The pixels neighboring a label are progressively invaded, beginning with the pixels of highest intensity, until a threshold has been reached. The watershed algorithm allows one to distinguish between the adjacent regions, as long as they present distinct maxima. In our current study, h-maxima were used to avoid over-segmentation of hair regions due to multiple local maxima. Local maxima are separated from each other by saddles, with h-maxima in particular representing a subset of local maxima with saddle intensity of at least h. The implementation efficiently integrates the identification of h-maxima and watershed segmentation into one algorithm [30]. Watershed stopping criteria and the parameters for selecting h-maxima were determined manually for a group of test focus stacks. To remove the background regions with low acquisition noise, the stop criterion was set at the background intensity value+ 2 standard deviations. The h-maxima parameter was related to the height difference between the intensity maxima. Its value was therefore a fraction, e.g. 10%, of average maxima height.

Each hair region detected by the segmentation was characterized by a vector. The high intensity of the hair root allowed us to define the hair origin as the position of each region’s intensity maximum. Orientation and length were deduced from each region’s covariance (i.e. the pixel position covariance). Orientation was given by the first eigenvector of the region’s covariance, and length was defined by the square root of that first eigenvalue. We ensured that the eigenvector was oriented from root to tip by making the coordinate sign for the eigenvector’s scalar product with the vector going from the region’s origin to its center of mass.

Once hairs were characterized, upper and lower wing hairs were separated based on their angle and root position relative to the wing surface. Both of these features change sign at the surface crossing. If the surface is considered locally flat, the sign of the hair’s angle to the surface is the same as the sign of the hair vector’s z-coordinate. Although the wing surface is transparent and could not be visualized in the images, its altitude in the z-stack was deduced from the altitudes of hair roots (i.e. the z-coordinate of the hair origin position) which are attached to the wing’s surface. Thus, the wing surface altitude at each hair root position was estimated by averaging the neighboring altitudes in a cylinder with a radius equivalent to 2 × the inter-hair length (roughly 25 μm). Subtracting this estimate from hair root altitude allowed us to estimate hair distance to the wing surface. Hairs were assigned to the top or bottom side of the wing if both their angle and distance to the surface were positive or negative, respectively.

Finally, on each side of the wing, hairs were classified as having a wild-type or *mwh* phenotype, depending on the distance to the closest neighbor. Only hairs on the same side of the wing surface were considered when determining the closest neighbors. If the distance to the closest neighbor was less than 5 microns, a hair was considered to exhibit the *mwh* phenotype. The threshold distance is a fraction of the average distance between individual hairs. The latter was measured to be 12.5 ± 2.5 μm from the distribution of relative distance between hairs measures using all detected hairs. This distribution also clearly identifies a second group of hairs, corresponding to *mwh*, which was well selected using the 5 μm threshold.

### Discarding regions where proper hair detection could not be conducted

In parallel to the hair region identification, we performed a second image analysis, aimed at detecting regions where no proper hair analysis could be conducted. Sometimes a wing’s border and veins display hairs, but their morphology and density differ from those observed on the rest of the wing and should be omitted from the analysis. Similarly, regions outside of the wing should not be analyzed. An analysis based on the density of structures in the image was developed to distinguish areas of interest (medium-density) from veins (high-density) and outer wing regions (low-density). The original z-stack was first reduced to a 2D image with a maxima projection in which only the maximum intensity along each z-column of the image was kept to build the projection image. A threshold was applied to the projection to remove the background and define a structure mask. The local density of structures in the mask was measured by applying a Gaussian convolution with a standard deviation equivalent to 2 × the inter-hair distance to the mask. Low-density and high-density region masks were created by applying thresholds to define a discard mask. Additionally, detecting high-intensity regions required an initial normalization of the intensity range in the projection image, to correct for local heterogeneity in structure intensity. Hairs obtained from watershed segmentation with at least one pixel in the discard mask were discarded. Although the method was initially intended to detect veins and regions outside of the wing, it also coped well with partially folded or torn wings that were not detected or discarded in the earlier acquisition step.

### Discarding hairs with inconsistent parameters

Isolated errors in segmentation and hair characterization can significantly decrease the quality of *mwh* detection. Three complementary analyses were performed to discard errors in areas of interest. First, we assumed a common local orientation of hairs and discarded hairs for which the orientation was too dissimilar. To do so, we computed the scalar product of the target hair’s vector and that of its average neighbor and rejected hairs that yielded a negative value. Using the second approach, we estimated the distribution of simple hair shape descriptors and discarded hairs with abnormal descriptors. This method helped detect under-segmented objects, as described in detail in the S1 File. Finally, determining a hair’s position relative to the wing allowed us to detect hairs with an abnormal relative position or orientation to the wing surface. We discarded hairs with (1) a root distance to the surface greater than the average root altitude + 2 standard deviations;(2)ambiguous angles relative to the surface of the wing (i.e., angles less than10% of the average angle); or(3)an altitude and orientation to the surface with different signs (Fig 4B).

### Wing genotoxicity score calculation

A genotoxicity score was calculated for each wing with at least one focus stack. Scores can be calculated as (1) the number of *mwh* hairs per wing, normalized by the wing surface area analyzed; (2) the number of *mwh* cells per wing; (3) the number of *mwh* spots per wing; and (4) a dichotomous variable, indicating whether the wing displayed more than 2*mwh* hairs. In this study, we used the number of *mwh* cells per wing to assess the genotoxic effects.

### Characterization of test compound genotoxicity

The genotoxicity profile softest compounds were determined using dose-response curve (DRC) analysis. A DRC for each test compound was constructed by plotting the average wing scores measured at each dose. The presence of a genotoxic effect was assessed first by fitting a straight line to the DRC. A Student’s *t*-test was used to test the null hypothesis that the slope coefficient of the line of fit was less than or equal to zero. If the hypothesis was rejected (*P* <0.05), the slope was most probably positive, and the compound was considered genotoxic. Otherwise, it was considered non-genotoxic. We call this test the dose-dependency test, as it allows one to detect whether genotoxicity increases with dose, as would be expected. It is not obvious, however, that this assumption holds at toxic doses. Therefore, data points at which compounds showed toxicity were discarded prior to the analysis.

Compounds identified as genotoxic in the dose-dependency test were further analyzed by fitting a sigmoidal function to the data. Since genotoxic compounds can become toxic at high concentrations, our measures did not include the final, saturated section of the sigmoidal curve. To compensate for this lack of information, a saturation value was enforced during curve fitting. This allowed us to deduce three parameters: the minimum effect value, the EC_50_, and the so-called slope parameter. The fit parameters and their confidence interval were estimated using MATLAB functionsnlinfit and nlparci, available from the statisticstoolbox.Acurve-fittingenvelopewassubsequentlyestimated using the fit parameter covariance and the confidence interval. Details on parameter interpretation and choice of the sigmoidal curve range are provided in the S1 File.

## Supporting Information

S1 File Supplementary information: the file contains additional methodological details describing the image analysis procedure, the genotoxic scores tested, and the MatLab package developed for the study. (DOCX)

S1 Table Genotoxicity scores per dose: the table contains averaged genotoxicity scores, presented for each compound, at each tested dose. The results were obtained with the MATLAB package provided.
(CSV)

S2 Table Genotoxicity scores per wing for automatic measure: the table contains genotoxicity scores of each of the 3,002 wings measured using the automated SMART process. (CSV)

S3 Table Genotoxicity scores per wing for manual measure: the table contains genotoxicity scores measured manually in the slides, which were also analyzed using the automated approach. (CSV)

S4 Table Result of the dose-dependency test: the table contains the results (*P*-values) of dose-dependency tests for each compound and for each type of wing score assessed in the study. (CSV)

S5 Table Genotoxicity profile: the table contains the results of the fitting of the sigmoidal curve for genotoxic test compounds (i.e., *P*<0.05 in the dose-dependency test) for the different types of wing scores assessed in the study. (CSV)

S6 Table Result of the two-samples test: the table shows the results of two-sample tests comparing treatment data (for a given compound/dose) to control data. (CSV)
