# Supplementary material for: High-Throughput In Vivo Genotoxicity Testing: An Automated Readout System for the Somatic Mutation and Recombination Test (SMART)
Source: PLoS One. 2015 Apr 1;10(4):e0121287. doi: 10.1371/journal.pone.0121287 (PMC4382174; doi:10.1371/journal.pone.0121287)
Supplement: S1 File — (DOCX) [file pone.0121287.s001.docx]

# High-throughput, *in vivo* genotoxicity testing: an automated readout system for the Somatic Mutation And Recombination Test (SMART)

Benoit Lombardot^1^, Chun-Taek Oh^2^, Jihoon Kwak^1^, Auguste Genovesio^1^, Myungjoo Kang^3^, Michael Adsett Edberg Hansen^1^, Sung-Jun Han^2^

^1^Image Mining Group, ^2^Drug Biology Group,Institut Pasteur Korea, Sampyeong-dong 696, Bundang-gu, Seongnam-si, Gyeonggi-do, Korea.^3^Department of Mathematical Sciences, Seoul National University, 1 Gwanak-ro, Gwanak-gu, Seoul 151-747, Korea.

**Supplementary Materials**

Contents

Additional Methodological Details 2

Experimental conditions and assay optimization 2

Slide preprocessing for image acquisition 2

Optional mask filtering 2

K-means clustering principle 3

Detecting slide position and orientation 3

*mwh* hair detection 3

Detection of under-segmented hairs 3

Genotoxicity characterization 4

Wing score calculation 4

Sigmoid fitting 4

Confidence interval and sigmoid envelope calculations 5

Results: Additional Data 6

Percent hatching data for six test compounds 6

Genotoxicity wing scores measured 6

Detecting genotoxicity with the two-sample test vs. the dose-dependency test 7

Comment on the sensitivity of different types of wing scores 7

Genotoxicity detection: manual vs. automated measures 8

Genotoxicity profiles: manual vs. automated measures 8

Genotoxicity profiles obtained using various types of wing scores 9

The false positive rate in automated *mwh* detection 10

Supplementary Data 12

Custom code organization and usage 12

Code for slide preprocessing 13

Code for *mwh* hair detection 14

Code for genotoxicity profile characterization 16

Use of external code 18

Data provided with the paper 18

Slide image samples 18

FocusStack_Samples 18

Mwh detection – MMS Result 18

Data supporting genotoxicity analysis: Paper_Results_genotoxicity 19

# Additional Methodological Details

## Experimental conditions and assay optimization

Very often, insoluble chemicals are dissolved in DMSO,which could result in toxic effects independent of the chemical of interest (Graf et al. 1984)**.** To determine proper conditions for DMSO usage, we performed several tests (Supplementary Fig. 1**)**. When 3-day-old larvae were exposed to DMSO solution for 24 hours, we observedtoxic side effects at concentrations as low as 2%;by contrast, a 6-hour exposure did not result in any significant damage at concentrations up to 16%. Treatment with a high concentration of DMSO resulted in severe, genotoxic effects. Based on these findings, we decided on these feeding conditions: 6 hours with 10% DMSO solution. To reduce the numberof phenotypes to analyze and thus facilitate more robust image analysis, we simplified the genetic basisof the SMART by using a fly strain carrying a single copy of the *mwh* mutation (rather than both the *mwh* and *flr* mutations, as in the original SMART approach).

## Slide preprocessing for image acquisition

### Optional mask filtering

The mask obtained after applying an image threshold to the scanned image can be filtered using two mathematical morphology operations: a closing filter to regularize objects, followed by an opening operation to separate them. The closing operation aims to remove holes and regularize the contours of the mask. The goal of the subsequent opening operationis to separate the parts of the mask that correspond to different objects; otherwise, the labeling algorithm will not always be able to distinguish between them. The radius of the kernel used for each operation should be a fraction of that of the objects one wishes to identify. In particular, the radius of the opening kernel must be larger than the radius of the closing kernel, otherwise it will have no effect. Both filters can be used to improve the quality of the mask and thus object segmentation.


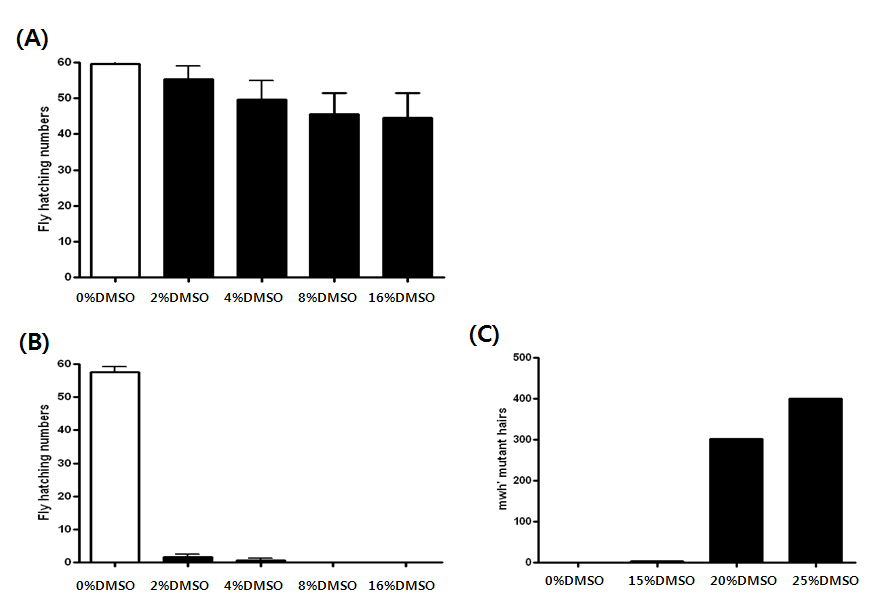


**Supplementary Figure 1.** Testing for toxic effects of DMSO. (A, B) Three-day-old larvae were fed chow containing different percentages of DMSO for either (A) 6 hours or (B) 24 hours.Later,the number of hatched flieswas counted. (C) Three-day-old larvae were fed chow containing different percentages of DMSO for 6 hours. Later, the number of *mwh* hairs on adult fly wingswas counted.

### K-means clustering principle

K-means clustering is a popular algorithm for clustering observations. It partitions a set of observations into N groups, each observation belonging to the cluster withthe closest average. Many heuristics exist foridentifying clusters. One of them involves: (1) randomly choosing N positions for cluster centers in the observation space, (2) assigning each observation to the closest cluster center (the association step), and (3) updating the center positions to reflect the average of the cluster observations (the updating step). The association and updating steps are iterated until the clusters converge. Here, the observations are the positions of the pixels belonging to an identified wing. Thus, the algorithm partitions the wing into N regions of compact shape.

### Detecting slide position and orientation

Microscope axes are aligned with the slide edges. Thus, detecting slide edges in scanned images allows one to define microscope referentials. Slide position can be determined manually for each sample in the image; however, it is also possible to determine it reliably using an automated method. Applying a threshold of 50% of the intensity range to the inverted scanned image allows the user to create a mask, in whicha slide’s long edges can be detected as the two most elongated regions. Prior to selection, each independent region’s length is defined by the largest eigenvalue ofthat region’s pixel position covariance. The average orientation ($\theta$) and position ($X_{c}$) of the two most elongated regions then define the slide’scenter and orientation.When employing manual detection, we used a simple custom code to identify the four corners of the slide in the image, deducing from them its orientation and position.

## *mwh* hair detection

### Detection of under-segmented hairs

The method described here helps detect under-segmented objects. It estimates the distribution of normal hair shape descriptors (volume, length, and the second eigenvalue of region covariance) and discards hairs with uncommon descriptors. This techniqueconsists of (1) estimating the 3D distribution of these parameters in each image using a Gaussian distribution and (2) discarding hairs far from the estimated Gaussian center.Estimation is performed iteratively and initialized with descriptor averages and variances. Prior to initialization, each parameter is centered and normalized according to the average and standard deviation of the 90% of observationsnearest the parameter median.With each iteration, the Gaussian position and covariance are re-evaluated by weighing each data point according to the distribution probability at that position. Furthermore, the covariance determinant is kept constant by rescaling ateach iteration. This avoids divergence of the estimation. The distribution is estimated with ten iterations for each wing sub-volume. The Mahalanobis distance, $d$, is calculated for each hair. For the i^th^ hair,$d$is given by:

$d_{i}=\sqrt{{}^{t}\left( X_{i}-X_{c} \right)\Sigma^{-1}(X_{i}-X_{c})}$.

Where $X_{i}$ is the i^th^ hair parameter vector and$X_{c}$ and $\Sigma$ are the distribution’s estimated center and covariance, respectively. Hairs with a $d$ greater than six are discarded. Though a threshold of three would have beenadequate to select more than 99% of well-segmented hairs, a higher threshold is preferable, since *mwh* hairs do not follow a Gaussian distribution and should not be discarded by too-stringent selection criteria.

## Genotoxicity characterization

### Wing score calculation

A wing genotoxicity score was calculated for each wing withat least one focus stack. There are multiple ways to calculate such scores: the number of *mwh* hairs per wing, normalized by individual wing surface area; the number of *mwh* cells per wing; the number of *mwh* spots per wing; or a dichotomous variable indicating whether the wing displayed at least k *mwh* hairs, cells, or spots, with k being a user-defined integer. In our study, genotoxicity characterization was based on the normalized number of *mwh* cells per wing. It was calculated as the ratio of (1) *mwh* hairs summed over the focus stacks associated with a wing and(2) the analyzed wing surfacearea,this latter definedas the fraction of focus stacks not discarded due to the presence of veins and outer wing regions. The resulting ratiowas multiplied by the average number of focus stacks per wing.

In order to count the number of *mwh* cells and spots per wing, the *mwh* hairs detected were grouped according to relative distance. To group hairs in cells or spots, we implemented a simple regiongrowing algorithm that works as follows. Given a list of *mwh* hairs and their neighbors, one *mwh* hair can be selected and assigneda label.As long as an *mwh* hair can be found within a distance,R, of another hair with that label, the group grows. When no other *mwh* hairs are in the vicinity of the group, growth stops and a new group can be initiated with *mwh* hairs not yet assigned a label. As each group is given a unique label, the number of groups can be easily counted. The choice of the neighborhood parameter, R, can be adjusted to gather *mwh* hairs belonging to the same cell or the same spot. If R is chosen to be equal to or less than the inter-hair distance, the group constructed will correspond to *mwh* cells. Alternatively, if R is chosen to be 1.5 times the inter-hair distance, the groups will correspond to spots visible in the focus stack.

### Sigmoid fitting

The sigmoid function used for curve fitting was parameterized as follows:

$$f\left( X \right)= A+\frac{B}{1+e^{\frac{C-X}{D}}}$$

The meaning of these parameters is illustrated in Figure 2. D remains positive when the sigmoid fitting is performed by enforcing the condition D = exp(d). X is equal to log2(c/c0), where c is the concentration of the tested compound.A, B, C, and D represent the bottom, the range, the EC_50_, and the slope, respectively.

Slope depending
on D


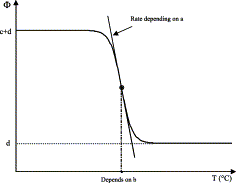


A+B

C

A

**Supplementary Figure 2**. Sigmoid model parameter illustration

### Confidence interval and sigmoid envelope calculations

The fitting function provided estimates for sigmoid best-fit parameters and their covariance. These data were used to estimate 95% confidence intervals with the function nlparci (MATLAB statistics toolbox).

To illustrate the influence of parameter variability, an envelopewas estimated for each of the sigmoid fittings performed. The best-fit parameter (P_0_) and its covariance (S_0_)were used to determine a set of parameters couples. Those with a Mahalanobis distance less than 1.96 (corresponding to a 95% confidence interval) were selected: ^t^(P-P_0_) S_0_^-1^ (P-P_0_) <1.96. The upper and lower edges of the envelopewere estimated by plotting the curve with the selected parameter couples, keeping the maximum and minimum of all curves at each sample position.

# Results:Additional Data

## Percent hatching data for six test compounds

Data points for highly toxic dosages (i.e. those for which more than 50% of *Drosophila* did not hatch) were omitted from further analysis, since they could lead to biased measures by selecting for less-exposed flies. In practice, only MMC concentrations>1.25 μM/L were discarded. Supplementary Table 1shows the percent of larvae hatchingfor the six compounds. Three replicates of 50 eggs each wereanalyzed for each independentcompound/concentration.

**Supplementary Table 1.**Percent of flies hatching after exposure to six test compounds.

|  | Antipyrin | | Atenolol | | Isoniazid | | MMC | | MMS | | Ur | |
| --- | --- | --- | --- | --- | --- | --- | --- | --- | --- | --- | --- | --- |
|  | Avg | SD | Avg | SD | Avg | SD | Avg | SD | Avg | SD | Avg | SD |
| 0μM | 0.92 | 0.05 | 0.93 | 0.01 | 0.95 | 0.01 | 0.93 | 0.03 | 0.94 | 0.02 | 0.92 | 0.02 |
| 39 μM | -- | -- | -- | -- | -- | -- | 0.93 | 0.03 | -- | -- | -- | -- |
| 78 μM | -- | -- | -- | -- | -- | -- | 0.94 | 0.02 | -- | -- | -- | -- |
| 156 μM | -- | -- | -- | -- | -- | -- | 0.92 | 0.03 | -- | -- | -- | -- |
| 312 μM | 0.93 | 0.03 | 0.93 | 0.03 | 0.96 | 0.03 | 0.91 | 0.01 | 0.91 | 0.01 | 0.93 | 0.01 |
| 625 μM | 0.95 | 0.02 | 0.94 | 0.02 | 0.93 | 0.01 | 0.88 | 0.02 | 0.90 | 0.02 | 0.93 | 0.03 |
| 1.25 mM | 0.93 | 0.03 | 0.95 | 0.04 | 0.90 | 0.02 | 0.67 | 0.03 | 0.87 | 0.03 | 0.92 | 0.03 |
| 2.5 mM | 0.91 | 0.01 | 0.95 | 0.01 | 0.95 | 0.01 | 0.1 | 0.04 | 0.85 | 0.03 | 0.93 | 0.03 |
| 5 mM | 0.95 | 0.03 | 0.92 | 0.03 | 0.93 | 0.03 | 0.03 | 0.01 | 0.75 | 0.02 | 0.93 | 0.03 |
| 10 mM | 0.91 | 0.02 | 0.91 | 0.01 | 0.92 | 0.04 | 0 | 0 | 0.53 | 0.06 | 0.92 | 0.02 |
| 20 mM | 0.91 | 0.01 | 0.92 | 0.02 | 0.90 | 0.02 | -- | -- | 0.05 | 0.02 | 0.93 | 0.02 |
| 40 mM | 0.88 | 0.02 | 0.91 | 0.01 | 0.89 | 0.02 | -- | -- | 0.00 | 0.00 | 0.91 | 0.01 |

## Genotoxicity wing scores measured

Four types of wing scores were calculated from automated measures:

- **ws1:**The number of *mwh* hairs per wing.
- **ws2:**The number of *mwh* cells per wing.
- **ws3:**The number of *mwh* spots per wing.
- **ws4:**The fraction of wingsdisplaying more than two *mwh* hairs (a cut-off of two was chosen to lower impact of false positive detection).

Two types of wing scores were calculated from manual measures:

- **ws5:**The number of *mwh* cells per wing.
- **ws6:**The fraction of wings displaying more than zero *mwh* hairs.

## Detecting genotoxicity with the two-sample test vs. the dose-dependency test

For each of the six types of wing score studied, the average wing score for control sampleswas measured (Supplementary Table 2). The wing score for each compound/dose was then compared to the null expectation using a two-sample normal test (Supplementary File,twosampletest_Pval.csv).

In addition, a dose-dependency test was performed using each compound’s wing scores, as described in manuscript online methods section. The resulting p-values are shown in Supplementary Table 3.The null hypothesis, as well as an inverse dose-dependent relationship, could be rejected when p<0.05.

We found that dose-dependency testing was more sensitive to genotoxicity. Notably, it appeared that for the range of doses initially studied, from 0.3–10 mM, the two-sample test did not detect a significant difference from the control using either manual or automated measures.

**Supplementary Table 2.**Average wing scores in control samples, with standard errors.

|  | ws1 | ws2 | ws3 | ws4 | ws5 | ws6 |
| --- | --- | --- | --- | --- | --- | --- |
| Controlaverage | 2.46 | 1.17 | 0.98 | 0.11 | 0.17 | 0.04 |
| Control standard error | 0.37 | 0.17 | 0.12 | 0.13 | 0.08 | 0.08 |

**Supplementary Table 3.**P-values obtained from wing score dose-dependency testing.

| Compound | ws1 | ws2 | ws3 | ws4 | ws5 | ws6 |
| --- | --- | --- | --- | --- | --- | --- |
| MMC | 0.0014 | 0.0015 | 0.0006 | 0.0102 | 0.0030 | 0.0050 |
| MMS | 0.0038 | 0.0038 | 0.0026 | <0.0001 | 0.0062 | <0.0001 |
| Urethane | 0.0029 | 0.0032 | 0.0264 | 0.2756 | 0.0026 | 0.6453 |
| Isoniazid | 0.4963 | 0.6620 | 0.7858 | 0.9852 | 0.3583 | 0.1572 |
| Antipyrine | 0.3314 | 0.3548 | 0.3090 | 0.5890 | 0.7760 | 0.2502 |
| Atenolol | 0.8698 | 0.8870 | 0.8759 | 0.9309 | 0.6911 | 0.8043 |

## Comment on the sensitivity of different types of wing scores

Measuring the fraction of wings with two or more *mwh* hairs was not sensitive enough to detect the low level of genotoxicity displayed by Ur; this was true at all concentrations tested, for both manual and automated measures, and for both the two-sample and dose-dependency tests. Of the remaining measures, *mwh* hair and cell counts seemed to give the best results based on p-values, as seen in Supplementary Table 3andthe file **twosampletest_Pval.csv**. Cell count-based wing scores (ws2 and ws5) seemed best suited for performing genotoxicity characterization, since both the manual and automated measures proved sensitive with few false detects.

## Genotoxicity detection: manual vs. automated measures

Manual and automated measurements yielded similar two-sample test results.For example, MMC and Ur genotoxicity were detected at the same concentrations for both manual and automated measures. However, MMS genotoxicity was detected at a lower doseusingmanual measurements, for various types of wing scores. As mentioned earlier, both automated and manual measures failed to detect Ur genotoxicity(1) using the two-sample test and (2) at the original, 0.3–10 mM range of concentrations tested.

When the dose-dependency test was used, manual and automated measurements identified genotoxicity for the same doses/compounds. In particular,Urtoxicity was detected at the initial range of concentrations tested, 0.3–10 mM. In addition, the test correctly identified non-genotoxic compounds. This demonstrates that the sensitivity of the dose-dependency test is superior to that of the two-sample test. Furthermore, the dose-dependency test summarized the results from all doses tested in one statistic. This allows the user to avoid the ambiguous results generated by the two-sample test; for example, using the two-sample test, Ur’s ws4 wing scores display a genotoxic effect at 20 mM that disappears at 40 mM.

## Genotoxicity profiles: manual vs. automatedmeasures

Direct comparison of manual and automated wing scores could not be performed due to protocol differences (e.g., wing score types, wing surface area analyzed, error rates)For instance, the manual approachallowed us to systematically analyze the entire wing surface area, whereas the automated approach analyzed, on average, 66% of the wing. These factorsrequire scaling and offsetting the automated measurements with respect to the manual measurements.The sigmoid curve is already well-parameterized for such an operation as the bottom and the range parametersaccount for the offset and scaling of the curve, respectively. Also the two remaining parameters, slope and EC_50_ will carry information on genotoxic effects that will be comparable for manual and automatic measurement.

Since no plato is observed in genotoxicity effect, range is a free parameter in sigmoid model andits value has to be fixed. In particular it can be chosen to compensate forthe scaling between manual and automated genotoxicity wing scores. For manual data the sigmoid range parameter must be chosen larger than the maximum effect observed. For automated data, one can choose the manual measure range parameter value divided by the estimated manual-to-automated-measure ratio,calculated in the least square sense. In practice,the range parameter for ws5 wing scores was set to 125% of the maximum ws5 score observed. Although this value can be chosen freely, once chosen it must remain static, since any change willalter the value of the curve fit parameter. Ws1, ws2, and ws3 range parameter for the sigmoid fitting were deduced as described. The range parameters defined for ws1, ws2, ws3, and ws5 can be used to create a genotoxicity profiles for new compounds, requiring no manual measurements. However, reference range parameter value reliability could be improved by enlarging the set of reference compounds used for evaluation. If a new method for wing score calculation or image analysis isintroduced, the range parameter calculationsmust be updated; however,these calculations can be based on the same wing focus stacks, thus avoiding the need to perform a new round of image acquisition.The ws4 and ws6 wing score range parameters can simply be set at one, their maximum value. Supplementary Table 4shows the parametersfor the six test compounds.

**Supplementary Table 4.**Range parameters used for the sigmoid fitting of wing score dose-response curves.

| Wing score | ws1 | ws2 | ws3 | ws4 | ws5 | ws6 |
| --- | --- | --- | --- | --- | --- | --- |
| Range parameter | 233 | 103 | 30 | 1 | 208 | 1 |

## Genotoxicity profiles obtained using various types of wing scores

**Supplementary Table 5A.**EC_50_ and slope parameters for each compound identified as genotoxic in the dose-dependency test, derived from a sigmoid curve fitting. Rows are sorted by wing score type.

| Compound | Wing score type | EC_50_ in mM | [EC_50_ 95% CI, lower bound ; | EC_50_ 95% CI, upper bound | Slope | Slope 95% CI, lower bound | Slope 95% CI, upper bound |
| --- | --- | --- | --- | --- | --- | --- | --- |
| MMC | ws1 | 0.74 | 0.50 | 1.10 | 1.15 | 0.69 | 1.91 |
| MMS |  | 24.62 | 22.91 | 26.46 | 1.03 | 0.96 | 1.11 |
| Urethane |  | 1.19x10^4^ | 26.17 | 5.39x10^6^ | 0.35 | 0.12 | 1.06 |
| MMC | ws2 | 0.75 | 0.51 | 1.11 | 1.14 | 0.68 | 1.91 |
| MMS |  | 24.23 | 22.78 | 25.78 | 1.03 | 0.97 | 1.10 |
| Urethane |  | 1.14x10^4^ | 23.47 | 5.53x10^6^ | 0.36 | 0.12 | 1.08 |
| MMC | ws3 | 0.86 | 0.53 | 1.38 | 0.88 | 0.52 | 1.51 |
| MMS |  | 20.81 | 17.50 | 24.75 | 0.97 | 0.79 | 1.18 |
| Urethane |  | 191.50 | 44.41 | 825.82 | 1.11 | 0.45 | 2.76 |
| MMC | ws4 | 0.23 | 0.14 | 0.38 | 2.85 | 0.93 | 8.74 |
| MMS |  | 4.99 | 3.32 | 7.49 | 0.82 | 0.59 | 1.14 |
| MMC | ws5 | 0.75 | 0.67 | 0.86 | 1.74 | 1.37 | 2.20 |
| MMS |  | 20.95 | 20.05 | 21.89 | 1.28 | 1.21 | 1.35 |
| Urethane |  | 681.23 | 345.21 | 1344.32 | 0.68 | 0.54 | 0.86 |
| MMC | ws6 | 0.24 | 0.21 | 0.27 | 3.97 | 2.70 | 5.84 |
| MMS |  | 3.24 | 2.86 | 3.67 | 1.02 | 0.92 | 1.13 |

**Supplementary Table 5B.**EC_50_ and slope parameters for each compound identified as genotoxic in the dose-dependency test, derived from a sigmoid curve fitting. Rows are sorted by test compound, to ease comparison between wing score types.Compound

| Compound | Wing score type | EC_50_(mM) | EC_50_ 95% CI, lower bound | EC_50_ 95% CI, upper bound | Slope | Slope 95% CI, lower bound | Slope 95% CI, upper bound |
| --- | --- | --- | --- | --- | --- | --- | --- |
| MMC | ws1 | 0.74 | 0.50 | 1.10 | 1.15 | 0.69 | 1.91 |
|  | ws2 | 0.75 | 0.51 | 1.11 | 1.14 | 0.68 | 1.91 |
|  | ws3 | 0.86 | 0.53 | 1.38 | 0.88 | 0.52 | 1.51 |
|  | ws4 | 0.23 | 0.14 | 0.38 | 2.85 | 0.93 | 8.74 |
|  | ws5 | 0.75 | 0.67 | 0.86 | 1.74 | 1.37 | 2.20 |
| MMS | ws6 | 0.24 | 0.21 | 0.27 | 3.97 | 2.70 | 5.84 |
|  | ws1 | 24.62 | 22.91 | 26.46 | 1.03 | 0.96 | 1.11 |
|  | ws2 | 24.23 | 22.78 | 25.78 | 1.03 | 0.97 | 1.10 |
|  | ws3 | 20.81 | 17.50 | 24.75 | 0.97 | 0.79 | 1.18 |
|  | ws4 | 4.99 | 3.32 | 7.49 | 0.82 | 0.59 | 1.14 |
|  | ws5 | 20.95 | 20.05 | 21.89 | 1.28 | 1.21 | 1.35 |
|  | ws6 | 3.24 | 2.86 | 3.67 | 1.02 | 0.92 | 1.13 |
| Urethane | ws1 | 1.19x10^4^ | 26.17 | 5.39x10^6^ | 0.35 | 0.12 | 1.06 |
|  | ws2 | 1.14x10^4^ | 23.47 | 5.53x10^6^ | 0.36 | 0.12 | 1.08 |
|  | ws3 | 191.50 | 44.41 | 825.82 | 1.11 | 0.45 | 2.76 |
|  | ws5 | 681.23 | 345.21 | 1344.32 | 0.68 | 0.54 | 0.86 |

## The false positive rate in automated *mwh* detection

The false positive rate associated with automated *mwh* hair detection was measuredusing two data sets: (1) control samples and (2) MMS-exposed samples tested over six concentrations. Measurementswerecarried out by superimposing hair vector characterizationsonto the maxima projection of the focus stack. Hairs detected as *mwh* vectors appeared red, while others appearedgreen. As a result, false positives could beeasily counted among all the *mwh* hairs detected. The results are presented in Supplementary Figure 3 and supplementary table 6**.** The *mwh* false positive rate in controls was 0.43 per wing, with a standard deviation of 0.24.

The MMS dose-dependency test showed that the null hypothesis (that there is no dose-dependency) could notbe rejected (Student’s t-test, p=0.14). To perform this test,false positive detection values over the various doseswere fitted with a line, and we asked whether the slope of the line was significantly different from zero or negative numbers.No significant difference was found. Additionally, the false positive rate measured at various MMS concentration is not significantly different from the false positive rate measured in control.

**Supplementary Table 6.**False positive rate as a function of MMS concentration.

| **Concentration** | **0.31 mM** | **0.62 mM** | **1.25 mM** | **2.5 mM** | **5 mM** | **10 mM** |
| --- | --- | --- | --- | --- | --- | --- |
| **FP rate (per wing, avg)** | 0.71 | 0.61 | 1.03 | 0.75 | 0.82 | 1.16 |
| **FP rate stdev** | 0.78 | 0.28 | 0.65 | 0.41 | 0.20 | 0.91 |
| **p-value(*)** | 0.34 | 0.26 | 0.16 | 0.21 | 0.07 | 0.18 |

(*) p-value obtained from a one-sided Student’s t-test with three degrees of freedom, comparing false positive rates for treatments with those for the control.


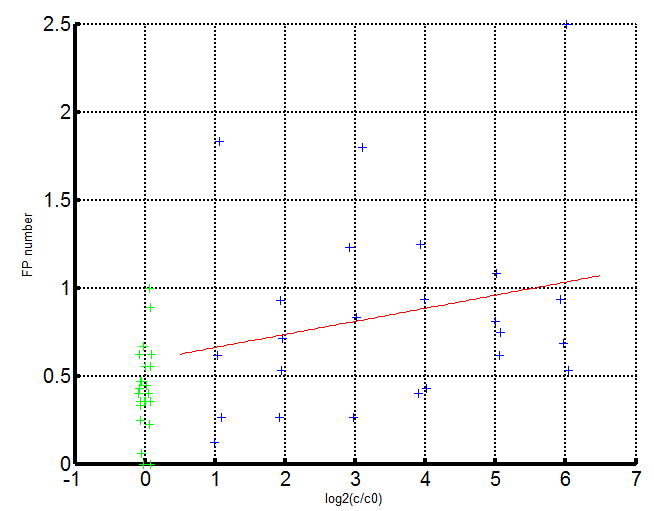


MMS concentration (Log2(c/c0))

False positive number per wing

control

**Supplementary Figure 3.**False positive rates in control slides (green crosses) and MMS-treated slides (blue crosses). Each cross represents the average number of false positives in a slide.

# Supplementary Data

Supplementary data are provided as a single archive named FW_supplementary_data_20140516.zip . Opening this .zip file will show the following directory structure:

- **Code**
  - **Automated_Acquisition**
  - **Autre**
  - **Common**
  - **Genotoxic_Analysis**
  - **Mwh_Detection**
  - **Outside code**
  - **Scripts**
- **Data**
  - **FocusStack_Samples**
  - **MMS - Mwh detection Result**
  - **Paper_Results_genotoxicity**
  - **Slide_Samples**

The “Code” directory contains the custom codes and scripts required to reproduce our analyses.The “Data” directory provides sample data from each important stepin the analysis; these data support our final results andillustrate the analytic process and its data structure.

## Custom code organization and usage

We distribute the custom code developed for the automated SMART along with this article. We also provide some scripts to illustrate the use of the code and some sample data with which the scripts can be used. The six scripts provided cover the three parts of analysis:

- Slide image preprocessing, prior to automatedimage acquisition
  - **FWAcq_processSlide2:** defines acquisition positions for automated microscopy
- *mwh* hair data extraction
  - **FW_detectMwhHair:** detects *mwh* hairs, cells, and spots in sample focus stacks
- Dose-dependent genotoxicity analysis
  - **FW_SaveAllWingData2:** saves all *mwh* detection results to csv files
  - **FW_scriptDisplayGenotoxicityAnalysis:** displays genotoxicity analysis results for a given compound.
  - **FW_profileCpd2:** performs genotoxicity analysis on all compoundsin the study and saves data.

Remarks:

- The code was tested on Windows 8 Professional and Windows 8.1.
- The code was tested using MATLAB2013a and 2010b.
- The code requires functions fromMATLAB’s image processing and statistics toolboxes.
- The custom watershed implementation, compiled ina .dll file, works only on Windows computers.
- The nd2 to fpg file converter relies on a Nikon nd2 SDK file kindly provided by Nikon. The executable, compiled program will work only in the Windows environment.
- With the two previousexceptions (the watershed program and the nd2 file converter), the code should work ina Mac OS environment,provided that the separator symbols in the file path are changed from “\“ to “/“.Because the code has not been tested in the Mac OS environment, however, some problemsmayarise and the user runs it at his/her own risk.

### Code for slide preprocessing

Process to be performed by the user:

**FWAcq_processSlide2**: This is a script calling all the necessary functions to perform slide preprocessing prior to image acquisition.

- Determine the path that leads to the slide images (data will also be saved to this path).
- Execute the script by pressing “run“ in the editor or by typing the FWAcq_processSlides2 script name in the console and pressing enter.
- An image showing the slide will open, allowing the user topoint to the corner of the lamella(s) on the slide. One right click adds a point, and a double right click closes the polygon. After pressing “q”,a final polygon must be enteredbut will be discarded.
- Subsequently,the user will beasked to point to all the lamellas. When one point is finished,thatimage will close and the next willopen.
- When manual pointing to the lamellas is complete, the slideswill beprocessed andslide position and orientationwillautomatically be detected. Then, wings will besegmented and their origin and orientation defined. This process lasts a few seconds.
- Next,the slide image will beopened with a transparent overlay representing wing segmentation. Each segmented wing is represented with a distinct, random color. Wing origin and orientation are representedby a green point and segment, respectively. The user can drawa polygon which content will be removed (after pressing R) or added (after pressing A) to the segmentation while respecting existing segmentations and labels. Also, wing originscan be updated to current mouse pointer position by pressing P in a segmented area.
- Each wing region issuccessively processed to determine acquisition positions. In an optional step, the order of regionscan be rearranged to minimize travel distance between positions. Acquisition positionsare then converted to microscope referential and saved to an xml file that can be read bythe NIS Element software controlling theNikon microscope.
- Subsequently, for each slide image, images are created showing acquisition regions, in green, the path between regions, in yellow, and region numbers, in white. For each lamella,twoimages are created: one showing the entire slide and the other a close-up of the lamella analyzed. These two images are useful for the initialization of microscope acquisition, as they show where to position the microscope stage to start.

Custom-made functions for slide preprocessing

- **polygons = draw_polygon_2d(img)**
  Takes an image (2d array) and generatesa list of polygons. This function opens an image and allowsthe user to input polygon positionswith the mouse while simultaneously drawing polygons. Double clicking on a point finishes the polygon and starts the input of a new polygon. Pressing the ‘Q’key allows the user to quit the interface after finishing the current polygon.
- **slide = FWAcq_slideDetection(img, slide)**
  Thisfunction detects the slide’s center and orientation automatically. Img is the slide image, and slide is a structure containing information on the slide.
- **[imgWingwingFeat]= FWAcq_wingSeg2(img, slide, viewResult)**
  This function performs the detection and segmentation of wings. imgwing is a labeled image of wing segmentation, and wingfeatis a structure containing information on wing features. In each field of the structure, the elements at the ith position match with object labelled by the value i in imgWing.
- **[wingFeatimgWing]= FWAcq_CorrectWingSeg(img, wingFeat, imgWing)**Thisfunctionloads the user interface forcorrecting segmentation.
- **[pos2Acq regInfoimgClasses] = FWAcq_AcqRegionDefinition4(imgRes, slides, img, imgWing, wingFeat,wingSel)**This function partitions each wing intonparts or less according to wingsize. It receives a selection of labeled regions.imgRes, slides, img, imgwing, wingfeat and wingSelare microscope resolution, slide information, slide image, wing segmentation, wing feature and a selection of wing to process. It returns a list of 2d positions in slide image coordinates (pos2Acq) and an image showing individual wing partitions (imgClasses).
  Note: this function makes use of the function kmeans from the MATLAB statistical toolbox.
- **pos2Acq = FWAcq_orderStack(pos2Acq,nIter)**This function orders the acquisition positions determined in **FWAcq_AcqRegionDefinition4** to minimize the length of thepath visiting all of them.
- **data = FWAcq_prepare_data_forXMLwritting(pos2Acq, regInfo, slide,imgRes)**This function convertsacquisition positions from slide image referentials to microscope referentials, aggregating them with wing label information.
- **writeXMLMultipoint4NIS(data, xmlFileName, xmlFilePath1)**Thisfunction writes an XML file, with name xmlFileName and in path xmlFilePath1, that can be read by the microscope software (NIKON NIS Element).

### Code for *mwh*hair detection

Sample script to test the custom code developed for *mwh* hair detection

Before running the script, the file path (FW_projectRoot) should be set to the current location of the project, i.e. the folder containing the “\Data“ and “\Code“ directories.

- **Analyze a focus stacks**
  - **FW_detectMwhHair**: Unmodified, this script will analyze the sample focus stack file with fpg extension, provided in the “\Data\FocusStack_Sample“ directory. The results of the analyses for eachfocus stack will be saved in the same directory. Intermediary image processing results can be saved in “\Data\FocusStack_Samples\save_seg“.
  - After opening the script, the user can update the path tothe project‘s current location on the computer (FW_projectRoot).
  - The path to access and save data can be specified, as well as WSDLL flag (indicating wether to use the dll for watershed) and saveSeg indicating whether to save segmentation data.
  - The function can then be run. It will automatically perform segmentation of focus stacks, characterization of hairs, classification of *mwh* hairs, and counts for *mwh* cells and spots.
  - When this process is finished, the script will display a projection of the first focus stack, processed with an overlay showing vectors representing the detected hairs. Hair colors indicate whether they are discarded (blue), wild-type (green), or *mwh* (red). Pressing any key will trigger display of the next processed image.
- **Important data structures and parameters**
  - **fpgDir**: the directory where fpg files are stored.
  - **saveSegDir**: the directory where results data are saved.
  - **useWSDLL**: a flag indicating which implementation of the watershed algorithm will be used in the segmentation of the data.
  - **viewData**: a table that contains the projection of each focus stack processed; it is saved in fpgDir.
  - **hairFeat**: a table that containsa structure describing the hairs’ features in each focus stack.
  - **tag**: a table that contains a structure with a flag statingeach hair’s type (valid/unvalidor*mwh*/wild-type, for example).

Custom-made function for *mwh* hair detection

- **volInfo = readFPG2(expInfo.filenames{i}, fpgDir)**This function reads focus stacks saved in fpg format.
- **[volFilt, volSeg, maskROI] = FW_processImg_v3(vol,useWSDLL)**This function analyzes 3d images. If the useWSDLL variable is set to one, the function will use a custom-made implementation of watershed segmentation. This implementation is roughly50 times faster than MATLAB’s built-in watershed function.Vol is the volume to segment.
  - **volFilt = FW_volFilter(vol,r,sig)**This function performs background and noise removal for a focus stack.r and sig are the parameters for image top-hat filtering and Gaussian blurring of the image vol, respectively.
  - **volSeg = FW_segmentation0(volFilt,threshG,h,volmin)**If the useWSDLL variable is set to zero,this function is used to perform segmentation of hairs.VolFilt will be segmented with a starting seed defined by the h-maxima of parameter h and a stopping criteria threshold.
  - **Discardmask= FW_regionDetection2D_v2(volProj)**This function constructsa mask of the region in a focus stack to be discarded. VolProj is a maximum projection of the focus stack along the z-axis.
- **writeFPG(volInfo,savePath,saveName)**This function writes a 2d or 3d array to anfpg file.
- **hairFeat = FW_RegFeatures2(volSeg,volFilt)**This function calculates region-based features, with the labeled regions present in the segmentation. The function also detects the maxima of intensity in volFilt, to determine hair origin.
- **orient_sign = FW_orientHair2(hairFeat)**This function returns a table of 1 and 0. 1 if a hair is oriented similar to its neighbors and 0 otherwise.
- **[neigh, neighDist] = nn_getneigh3(List1,List2,rN,maxN,spac)**Thisfunction determines the neighbors, in List2, of each object in List1. rN is the maximum distance, and maxN the maximum number of neighbors. Spac is the dimensions of a voxel.
- **tag = FW_getSegOfInterest_v2(volSeg, maskROI, tag)**This function determines which hairs belong to the region of interest (i.e. not the veins or background) and stores this information in the variable tag.
- **tag = FW_classif_v6(hairFeat,tag,viewClassif,viewData)**This functionclassifies hairs as*mwh*or wild-type. It also discards hairs with improbable characteristics.
- **[Label, Size] = FW_findClusters(hairFeat,tag,dmax)**This function gathers *mwh* hairs in clusters, depending on the criterion dmax. Hairfeat and tag provide the necessary information about hair. The functionreturns twovariables:(1) Label, which gives the label of each cluster (the label is zero if a hair is not clustered) and (2) Size,whichgives the size of each cluster.
- **FW_display_mutantclassif(tag,hairFeat,volProj,vizType)**This function is a tool for visualizing the results of hair detection characterization and classification, focus stack by focus stack.It allowsthe user to visualize *mwh*/wild-type/discarded hairs and upper/lower wing hairs in different colors

### Code for genotoxicity profile characterization

Sample script to test the custom code for genotoxicity analysis and to explore the paper’s data

Before running the script, the file path (FW_projectRoot) should be set to the current location of the project, i.e. the folder containing the “\Data“ and “\Code“ directories.

- **Process the tag file to create agenotoxicity wing score per wing and per dose**
  - **FW_SaveAllWingData2:** This script loads all the *mwh* hairs detected and calculates the genotoxicity scores per wing. All the data are saved to a csv file. Wing scores are also averaged by dose and saved to another csv file. This file is used for further genotoxicity analysis. Unmodified, the script will read the tag file provided in “\Data\MMS - Mwh detection Result“. These data correspond to the automatedMMS analysis, as describedin the paper. csvfiles are saved to the same directory and include:
    - GenotoxicScores_MM_All_perWing_Auto.csv
    - GenotoxicScores_MM_All_perWing_Manual.csv
    - GenotoxicScores_MM_All_perdose.csv
- **Analyze a compound’sgenotoxicity and display the results**
  - **FW_scriptDisplayGenotoxicityAnalysis:** This script displays one compound’s genotoxicity results. One can choose the compound and two genotoxicity scores to display, one automatedand one manual score, for instance.Unmodified, the script will analyze the data provided in the file “GenotoxicScores_perdose.csv” in the directory “\Data\Paper_Results_genotoxicity.” This file provides the genotoxicity scores, per dose, for the six compoundsanalyzed in the paper.
- **Systematic dose-dependency, two-sample test, sigmoid fitting for all test compounds, and all wing scores**
  - **FW_profileCpd2:** This script performs three analyses, allowing the user to compare the relative performance of the two-sample and dose-dependency tests in detecting genotoxicity. The results are saved in the directory “ \Data\Paper_Results_genotoxicity“in the following three files:
    - twosampletest_Pval.csv saves the p-valuesfromthe two-sample test; one test wasperformed for each (compound;dose;score) condition tested.
    - doseDependency_Pval.csv saves the p-valuesfromthe dose-dependency test, performed for each of the sixcompoundsanalyzed in the paper.
    - genotoxicProfile.csvsaves the sigmoid fitting data for the three compounds considered genotoxic based on the dose-dependency test.

Custom-made function for genotoxicity characterization

- **WingStat= FW_getWingStat(mwhDataPath, TestName)**This function reads all of the tag files generated by *mwh* hair detection and gathers them according towings. It generatesa structure providing the four automated genotoxicity score types for each wing.It also uses one txt file built manually thatgathers information about the various slidesanalyzed for that compound. One example of these files (MM_All.txt) can be found in project root “Data\MMS - Mwh detection Result”.
- **writeCSV2(savename, data, names)**This function creates a csvfile with a name and location indicated in savename. datais the 2D table saved in the file,and names is a cell array indicatingthe column namesfor data.
- **data = readAsciiTable(filename, separator)**This function is used to read csvfiles. The separator is set to a comma. datais an array with the same number of rows and columns as the table in the data csv file.
- **[X, Y] = FW_getCpdWingScores(data, cpd_Id, c0, conc_Range, score_type)**
  This function selects the data corresponding to a compound’s genotoxicity score fora given concentration range from the data array with all genotoxicity scores. X is an array of the log concentrations, and Y is an array of the values for the chosen compound and scores.
- **range = FW_rangeParamFromRef(data,wingscoreType1,c0)**
  This function uses manually collected data to determine the range used for sigmoid fitting of automatedscores. Range is an array giving the range value to use as a function of score type.
- **[p_val1, Pline, P, pRange, CovP, profile1] = …
  … FW_getCpdProfile(X, Y, data, c0, score_type, range)**This function performs further analysis based on a compound’s dose-dependent genotoxicity score. First, it measures whether the score is dependent ondose. If yes, it performs a sigmoid fitting of the data.
- **[p_val, Pline] = FW_isGenotoxic(X,Y)**This function tests whether Y is dependent on X, using the function polyfit3 to perform a weighted line fitting of the data. It returns the p-value of the statistical test (there is no dependency if p>0.05) and the coefficient of the data line fitting. polyfit3 is distributed under a BSD license.
- **[P, pRange, CovP, profile] = FW_fit_genotoxic_cpd(X,Y,range,c0, bottom0)**This function fits the points (X,Y) with a sigmoid curve of range “range.” c0 is used to deduce the EC_50_ from the sigmoid fit parameter, and bot0 is used to initialize the sigmoid fitting. P is an array with the fitted parameters (slope, EC_50_, and bottom). pRange gives their 95% confidence intervals. covP is the covariance of the sigmoid fit parameter, and profile containsthe actual EC_50_ and slope values withtheir confidence intervals.
- **hlist = FW_displayGenotoxAnalysis(X, Y, p_val, Pline, P, pRange, CovP, range, color, markerType)**This function displays the data in MATLAB, with Y as a function of X as well asits line or sigmoid fitting, depending on the P_val value. In the case of a sigmoid fitting, the function also draws a sigmoid envelope, according to the sigmoid parameter covariance given by CovP.
- **[avg, std] = FW_getControl(data, c0, score_type )**This function measures the average genotoxicity score and its standard error for control slides. Avg is an array giving the average value of the control slides for each type of genotoxic score.
- **pval = get_Student_OneSidedPval(t,df)**This function returns the p-value for the random variable t following a Student distribution with df degrees of freedom.

### Use of external code

- The function polyfit3 is available under afreeBSD license. The original code can be found here: <http://www.mathworks.com/matlabcentral/fileexchange/4262-polyfit3>
- The custom code makes use of the MATLABstatistical toolbox (nlinfit, nlparci, and kmean) and image processing toolbox (imopen), as well as a few original functions (string manipulation, data saving/loading, and array manipulation).

## Data provided with the paper

### Slide image samples

Twoslides images are provided and can be analyzed with the script FWAcq_processSlide2. Theirfilenames are:

- **MMS 10mM 2012-05-08 002.tif**
- **MMS control 2012-05-16 015.tif**

As the names indicate, they correspond to fly wings exposed to 10 mMand 0 mM MMS.

### FocusStack_Samples

1focus stacks, corresponding to acquisitions performed on 10 mM MMS-exposed fly wings, are provided in this file. The file namesareas follows:

- **MM 10mM 2012-05-08_lam2_stack_XXX_YYY_ZZZ.fpg**

XXX indicates the stackID in the slide: its value is 001 for the stack provided; YYY indicates the wing ID number in the slide;and ZZZ indicates the stack ID inthe wing.

These files can be processed with the script FW_detectMwhHairtodetect *mwh* hairs, cells, and spots.

### Mwh detection – MMS Result

All the *mw*h detection files for MMS are provided. There are threefiles per slide analyzed. The file formats are as follows:

- **MM_All_X_Y_info.mat**: gathers information on the slide and the focus stack analyzed. Contains the variable exp_info.
- **MM_All_X_Y_tag.mat**: gathers all classification information for each detected hair; for instance: valid/unvalid, upper/lower side of the wing, *mwh*/wild-type. Contains the variable tag.

MM_Allprefix corresponds to the compound tested,In this case it is MMS; X is the concentration, Y is the number of the slide forthat concentration (usually a value between 1 and 4). There are typicallyfourslides per concentration tested.

The script FW_SaveAllWingData2 can use these data to create csv files with all genotoxicity scores per wing and per dose.

### Data supporting genotoxicity analysis: Paper_Results_genotoxicity

This folder contains sevenfiles:

- **Compound_Id_Name.xlsx**: contains the relationship between a compound’s name and IDnumber.
- **GenotoxicScores_perdose.csv**: contains all averaged genotoxicity scores, measured per compound and per dose. The results presented in thispaper rely on the numbers shown here and can be reproduced with the scripts FW_scriptDisplayGenotoxicityAnalysis and/or FW_profileCpd2. The wsX naming convention is a shortcut for designating the genotoxicity score type X.
- **GenotoxicScores_perWing_Auto.csv**: contains genotoxicity scores for each of the 3,002 wings measured using the automatedSMART process.
- **GenotoxicScores_perWing_Manual.csv**: contains genotoxicity scores measured manually forthe same slides analyzed using the automated approach.
- **doseDependency_Pval.csv**: contains the results (p-values) of dose-dependency testing foreach compound, for each type of wing score assessedin the study.
- **genotoxicProfile.csv**: contains sigmoid fitting data for genotoxic test compounds (p<0.05 in the dose-dependency test) for various wing score types.
- **twosampletest_Pval.csv**: shows the resultsof two-sample testscomparing treatment data(for a given compound/dose) to control data.

The three last files are generated by the script FW_profileCpd2.
